# From Leiden to Tel-Aviv University (TAU): exploring clustering solutions via a genetic algorithm

**DOI:** 10.1093/pnasnexus/pgad180

**Published:** 2023-06-01

**Authors:** Gal Gilad, Roded Sharan

**Affiliations:** School of Computer Science, Tel Aviv University, Tel Aviv 69978, Israel

**Keywords:** graph clustering, modularity optimization, community detection

## Abstract

Graph clustering is a fundamental problem in machine learning with numerous applications in data science. State-of-the-art approaches to the problem, Louvain and Leiden, aim at optimizing the modularity function. However, their greedy nature leads to fast convergence to sub-optimal solutions. Here, we design a new approach to graph clustering, Tel-Aviv University (TAU), that efficiently explores the solution space using a genetic algorithm. We benchmark TAU on synthetic and real data sets and show its superiority over previous methods both in terms of the modularity of the computed solution and its similarity to a ground-truth partition when such exists. TAU is available at https://github.com/GalGilad/TAU.

**Significance Statement**

Graph clustering—the grouping of nodes to densely connected clusters—is a fundamental problem in data science with numerous applications. State-of-the-art approaches to the problem are based on greedy steps that tend to get stuck in sub-optimal solutions. Nevertheless, it is notoriously difficult to consistently outperform these approaches. Here, we present a novel approach, Tel-Aviv University (TAU), that can efficiently explore the solution space and thus lead to better solutions. We show that TAU is a scalable approach that outperforms previous approaches both in terms of clustering quality and similarity to ground-truth clusters.

Clustering is a fundamental problem in all fields of science with a plethora of formulations and algorithmic approaches (1). In graph clustering, the input to the problem is a graph whose nodes represent elements and edges represent their pairwise similarities. The goal is to partition the nodes into groups, called clusters, such that elements within a group are highly similar, or well connected to each other, while elements in different groups are dissimilar or not connected.

One of the most popular approaches to graph clustering is based on the modularity function introduced by Mark Newman (2). Specifically, this function quantifies the quality of a suggested partition by comparing the number of edges that lie within its clusters to their expected number if the graph was randomly drawn from the collection of all graphs with the same degree sequence. While optimizing the modularity is NP-hard (3), a number of heuristic approaches were devised for it (2, 4, 5).

The Louvain clustering method (6) is one such very successful approach that iteratively employs a greedy algorithm for modularity optimization. Its simplicity and speed have made it a method of choice for graph clustering problems. A recent improvement of Louvain is the Leiden algorithm (7) that uses the Louvain partition as a guide to form connected communities with high modularity. Both methods are greedy in nature, hence fast yet prone to suffer from convergence to local optimum solutions.

Here, we demonstrate the shortcomings of a greedy approach and propose a novel method, TAU, that combines Leiden’s greedy optimization with a genetic algorithm to better explore the solution space. We show that TAU consistently identifies higher modularity partitions compared to Leiden, on both real and synthetic networks, and that the detected communities are in higher agreement with ground truth communities.

## Methods

We designed a genetic algorithm that leverages the Leiden algorithm to detect communities in an input graph through modularity optimization. The algorithm maintains a population of possible clustering solutions, where the initialization scheme and the genetic algorithm operators are designed to allow for greater exploration of the solution space compared to Leiden. After an initialization step where the initial individual partitions are generated, the algorithm iteratively performs the following steps: (i) optimization of each individual partition using Leiden, (ii) fitness evaluation and selection of individuals, (iii) crossover and mutation in the population, and (iv) immigration of new individuals to the population. We now describe these steps in detail, denoting the input graph by *G*.

### Overview of Leiden

Leiden (7) partitions an input graph into clusters by optimizing the modularity function. It consists of three steps: (i) greedy node movements between clusters, (ii) partition refinement, and (iii) graph aggregation. Initially, each node is assigned to its own cluster. In the first step, nodes are traversed in random order and each node is moved to the cluster that yields the largest increase in the global modularity value. In the second step, in each cluster in the partition, each node is assigned to its own sub-cluster. Then, nodes are moved randomly within clusters to sub-clusters that are connected to them, as long as the global modularity value does not decrease. In the third step, the graph is reduced by transforming the sub-clusters of the refined partition into nodes. The new nodes are assigned to clusters according to the unrefined partition. These steps are applied iteratively until each cluster consists of a single node after Step (i).

### Initialization

To initialize an individual (clustering) *i*, either the nodes or the edges (equal probability) of *G* are subsampled with fraction p_subsample∼U[0.2, 0.9], to get a subgraph $G_{i}$. We apply Leiden (with default parameters) on $G_{i}$ to obtain a partition $P_{G_{i}}$ to communities. In order to create an initial partition with respect to *G*, we add singleton communities to $P_{G_{i}}$ for each node of *G* that is missing from $G_{i}$. The resulting partition is the initialized individual. hird.

### Optimization

In each algorithmic iteration, every individual partition is optimized by applying the Leiden algorithm to it. Specifically, starting from the given partition, nodes are first moved to communities that yield the largest increase in modularity, until no such move is possible. The resulting partition *P* then undergoes Leiden’s refinement procedure: for each community, nodes that are connected to the rest of the community nodes are allowed to merge and form connected sub-communities, as long as modularity does not decrease. In a third step, the graph is reduced by transforming the communities of the refined partition into nodes. In a fourth step, the nodes of the reduced graph are assigned to temporary clusters according to *P* and the four steps are iterated until convergence.

### Fitness and selection

The fitness of an individual is its modularity value. Individuals are ranked from 1 (worst) to the size of the population *S* (best) and selected for crossover and mutation processes with probability $P\_selection=\frac{rank_{i}^{\;power}}{\sum_{\;j=1}^{S}rank_{j}^{\;power}}$. The *power* parameter enables us to adjust the strictness of the selection, i.e. the higher the value the more likely top-ranked individuals are to be selected. The s_elite distinct (Jaccard similarity <0.9) partitions with the highest fitness values proceed as-is to the next generation, to guarantee nondecreasing top fitness values (*elitist selection*).

### Immigration, crossover, and mutation

We perform immigration, crossover, and mutation in the population to produce the remaining individuals for the next generation. First, s_immigrants new individuals are initialized and added to the next generation. The remaining individuals (S−s_elite−s_immigrants) are offspring of the previous generation. To produce each offspring, we draw two partitions from the population with probability P_selection and cross them. The crossover operator is simply the overlap between the partitions, i.e. nodes that co-occur in a community in both partitions will be mapped to the same community in the offspring; otherwise, they will be mapped to singleton communities. For example, given two partitions of nodes A,B,C,D,E to {A,B,C},{D,E} and {A},{B,C,D,E}, the overlap would be the partition {A},{B,C},{D,E}.

After crossover is completed, mutations occur in the offspring. A mutation is either a random merge of two connected communities, or a split of a random community into two communities using Newman’s leading eigenvector algorithm (5), with equal probability. We note that although the crossover and split operators are not guaranteed to preserve connectivity, all communities in the resulting partition are connected. This is since the subsequent application of the Leiden algorithm to the resulting partition, even if it contains disconnected communities, will produce a new partition in which all communities are connected thanks to the refinement procedure (7).

### Parameter selection and stopping criterion

A population of S=60 individuals (clusterings) is maintained throughout the generations. The genetic algorithm has three main parameters: selection strictness (*power*), immigrants fraction ($p\_immigrants=\frac{s\_immigrants}{S}$), and elite fraction ($p\_elite=\frac{s\_elite}{S}$). In order to choose the parameter values, we performed grid search on a randomly generated synthetic graph with 50,000 nodes (see *Benchmark data*) and on the CAIDARouterLevel graph. We examined values 1,3,5,7 for *power*, values 0.05,0.1,0.2 for p_elite, and values 0.1,0.15,0.3 for p_immigrants. We found that TAU’s performance is robust within the tested range of parameter values (Supplementary Figs. S1 and S2), and so the algorithm’s parameters were set to middle range values that performed well: power=5, p_elite=0.1 and p_immigrants=0.15.

As a convergence criterion, we tested for Jaccard similarity >0.98 between the best partitions of consecutive generations. We stop the algorithm when this criterion is met for 10 consecutive generations.

### Implementation details

TAU is implemented in Python 3. We use igraph (8) implementation of Leiden for partition optimization and networkx (9) implementation of the LFR benchmark (10). The script was run concurrently on S=60 cores (Intel(R) Xeon(R) Gold 6238 CPU @ 2.10GHz) using Python multiprocessing pool module.

To produce Leiden’s results, we perform consecutive runs of igraph’s implementation of the algorithm (with default parameter values) using the partition identified in one run as a starting point for the next run, as suggested by Leiden’s authors (7). We refer to each of these runs as an iteration of Leiden. For each input graph, we first run TAU and compute its total run time (until the stopping criterion is met). Then, we let Leiden run iteratively for the same amount of time.

### Benchmark data

We benchmarked TAU against Leiden using three sets of graphs. The first set is a collection of nine real networks that were gathered from Stanford’s SNAP collection (11) (Email, DBLP, Amazon, Youtube, Wiki, LiveJournal, all from the ground-truth section), Newman’s network data (12) (Astro-ph, As-22july06), and CAIDA (13) (CAIDARouterLevel). Three of the SNAP networks were also analyzed in the Leiden paper (DBLP, Amazon, and LiveJournal), and we complemented them by three additional networks for which approximate node labeling (termed “ground truth”) is available. Two of the networks (Email and Wiki) were directed, hence we focused on their undirected portions (53% and 12%, respectively) that contain only bidirected links. The last three networks (Astro-ph, As-22july06, and CAIDARouterLevel) were part of the DIMACS Graph Partitioning Challenge (https://www.cc.gatech.edu/dimacs10/archive/clustering.shtml) and are some of the more popular real-world networks for community detection benchmarking.

The second set includes two protein–protein physical interaction networks that were downloaded from STRING (14) (version 11.5): *Homo sapiens* and *Saccharomyces cerevisiae*. We binarized the networks and did not consider their interaction scores in our analysis.

The third set is a collection of synthetic graphs that were generated using the LFR approach (10). To generate these graphs, we used the implementation from networkx Python package and set the parameter values to the rounded average values from our collection of real networks (discarding the two largest networks): 288,535 nodes, average node degree 10, and an exponent of 3 for degree distribution (which was estimated using the powerlaw (15) Python package). We tested a range of four values for the μ parameter—the fraction of inter-community edges incident to each node—between 0.3 and 0.6 with 0.1 increments, and four values for the τ parameter—the exponent for the community size distribution—between 1.2 and 1.5 with 0.1 increments.

## Results

**Fig. 1.**
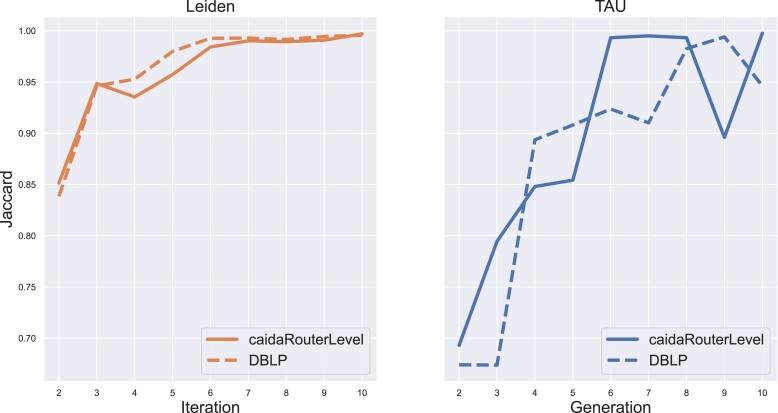
Partition similarity throughout Leiden’s and TAU’s optimization on CAIDARouterLevel and DBLP graphs (see Benchmark data section). Each point refers to the Jaccard similarity between the resulting partitions at iterations *i* and i−1.

We designed an efficient algorithm for modularity optimization that combines Louvain’s and Leiden’s greedy optimization with a genetic algorithm to better explore the solution space. To motivate our approach, we first study the behavior of Leiden over consecutive iterations by measuring the similarity between its solutions at consecutive iterations. As Fig. 1 demonstrates, the partitions produced by Leiden do not substantially change between iterations, indicating that the algorithm tends to be trapped in a local optimum. Since partitions that have similar modularity values can be significantly different from one another (16), Leiden’s inherent lack of solution space exploration likely limits its ability to detect better solutions.

In contrast, TAU seems to explore well the solution space by optimizing a population of different possible partitions. The TAU panel in Fig. 1 shows that the change in TAU’s solution across iterations is greater than Leiden’s and that even when an extremely fit individual is found (e.g. the plateau in generations 6 to 8 of CAIDARouterLevel), it can still be replaced by a better individual (clear dip in generation 9). To further support this observation, we computed the Jaccard similarity between all pairs of individuals in the first-generation population (after initialization and optimization of individuals, on CAIDARouterLevel graph). The average Jaccard similarity was 0.55, which indicates that our initialization scheme effectively yields distinct starting points for the optimization.

**Fig. 2.**
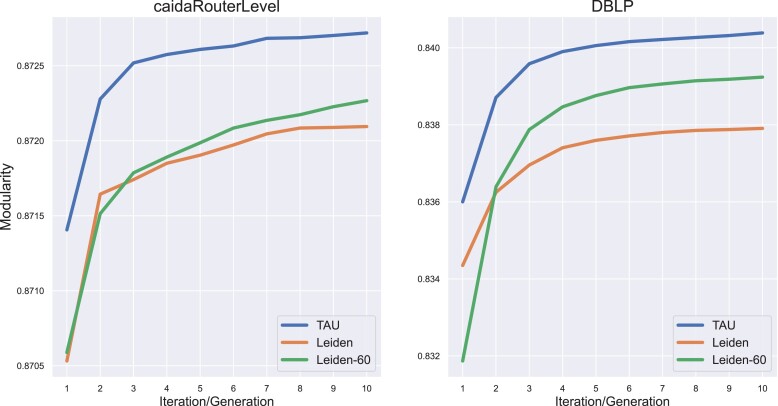
Partition modularity throughout Leiden-60’s and TAU’s runs on CAIDARouterLevel and DBLP graphs (see *Benchmark data*).

A simple approach to improve Leiden’s performance, which was used by its authors in the original paper, is to run it multiple times and choose the best-performing run. Since Leiden traverses the graph nodes randomly, each run is likely to converge to a different local optimum and yield a different partition. To examine this approach, we executed Leiden 60 times (referred to here as the *Leiden-60* approach). In each run, we performed 10 iterations, where we use the partition identified in one iteration as a starting point for the next iteration. Supplementary Fig. S3 shows the distribution of modularity values for the 60 resulting partitions on CAIDARouterLevel and DBLP graphs. While an increase in performance is observed, in both cases the top-performing runs offer only a limited improvement to the single Leiden run compared to TAU (Fig. 2). In addition, we computed the average Jaccard similarity between all pairs of CAIDARouterLevel partitions after the first iteration and found that it was higher than TAU’s (0.592±0.045 versus 0.548±0.041), further supporting the effectiveness of our initialization scheme. Similar improvements of TAU versus Leiden-60 can be observed across all other real networks (Supplementary Table S1 and Supplementary Fig. S4).

### Synthetic graphs

**Fig. 3.**
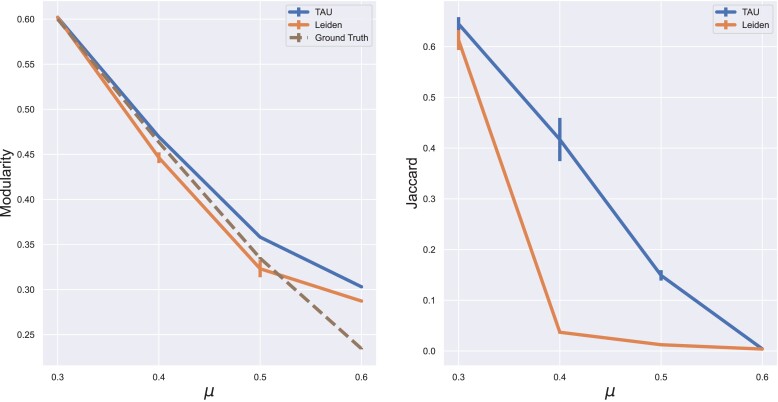
Comparative assessment on synthetic data. Each point is the average value (modularity or Jaccard similarity) over four different graphs that correspond to the four tested τ values (see Benchmark data section).

In order to benchmark our algorithm, we first tested in on synthetic graphs and compared to the state-of-the-art Leiden method. These graphs allow us to also examine the Jaccard similarity between the resulting partition of each method and the ground truth clustering. The results are summarized in Fig. 3.

TAU consistently uncovers better partitions compared to Leiden, both in terms of modularity and similarity to ground truth communities, across different values of the noise parameter μ and different community size distributions (controlled by τ). The improvement in modularity over Leiden is greater for μ≥0.4, where the partition to communities is more challenging. Interestingly, in terms of similarity to ground truth communities, the improvement peaks at middle-range values, μ=0.4 and μ=0.5. For μ=0.4, the Jaccard similarity between TAU’s partition and the ground communities is approximately 0.43, compared to only 0.04 for Leiden.

The community size distributions that arise from the reported τ values have little effect on the results. This effect is demonstrated by the error bars in Fig. 3. However, as we increase τ further (τ>1.6), we begin to observe a decrease in correlation between the modularity and the Jaccard similarity to ground-truth communities. This is likely due to the manifestation of the well-known resolution limit (17) of modularity, as there are more small communities in the synthetic graph as τ grows.

### Real networks

**Fig. 4.**
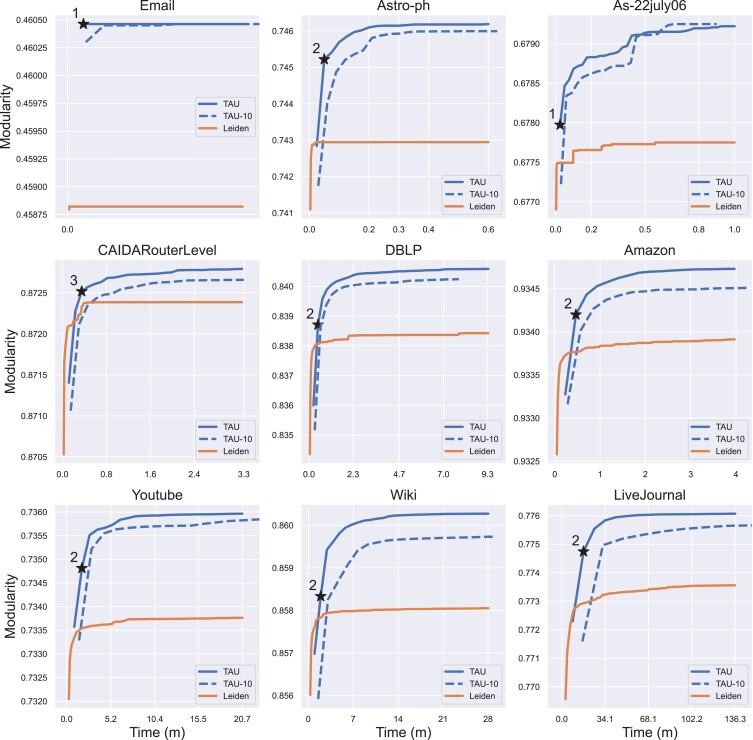
Comparative assessment on real data. The first generation where TAU finds a partition of higher quality compared to the final Leiden partition is marked with a star and the generation’s number.

Next, we evaluated the performance of TAU against Leiden on nine real-life graphs that were collected from three different sources. The results, summarized in Table 1 and Fig. 4, show that TAU consistently finds partitions of higher modularity compared to Leiden. Moreover, within the first three generations, TAU finds a partition that outperforms Leiden, even when the latter is run for hours (Fig. 4). Notably, TAU resulting partition can be quite different from Leiden’s solution, with Jaccard similarity as low as 0.35 (mean=0.58, std=0.09 over these nine graphs).

**Table 1.** Performance on real data.

Graph	Nodes	Edges	Modularity			*T*		
			TAU	TAU-10	Leiden	TAU	TAU-10	Leiden
Email	776	8,865	0.460462	0.460462	0.458822	0	0	0
Astro-ph	16,706	121,251	0.74619	0.745996	0.742948	1	1	0
As-22july06	22,963	48,436	0.679222	0.679249	0.677751	1	1	0
CAIDARouterLevel	192,244	609,066	0.872794	0.87266	0.872388	7	9	1
DBLP	317,080	1,049,866	0.840592	0.840248	0.838428	14	18	3
Amazon	334,863	925,872	0.93473	0.93451	0.933913	14	17	3
Youtube	1,134,890	2,987,624	0.735965	0.73588	0.733763	54	88	13
Wiki	1,281,369	2,988,992	0.860274	0.859733	0.858052	58	92	14
LiveJournal	3,997,962	34,681,189	0.776072	0.775741	0.77356	511	967	173

Modularity of partitions produced by TAU and Leiden. *T* is the average run time (rounded to whole seconds) per generation or iteration for TAU and Leiden, respectively. TAU-10 is a run with S=10 individuals, on a dual-core CPU.

To assess the consistency and statistical significance of TAU’s improvement over Leiden, we evaluated its performance against the multiple-run Leiden-60 approach. We executed Leiden-60 and TAU 10 times on each of the nine networks, and found that in 8 of the 9 networks, all 10 TAU runs outperformed all 10 Leiden-60 runs (P<0.00016 for each of these 8 networks by a Wilcoxon rank sum test; in the ninth network, both methods attained similar modularity values but the difference was still significant, P<0.0006). The results are summarized in Supplementary Fig. S4.

Furthermore, in order to demonstrate the relevance of our method to PC users, we evaluated the performance and run time of TAU when it is run with a population of S=10 individuals (TAU-10) on a dual-core CPU (Fig. 4, Table 1). The results show that this variant has comparable performance and run time.

The evaluation of TAU’s performance on synthetic data suggested that the improvement over Leiden may be more substantial for noisier networks, i.e. networks with larger fractions of cross-community connections. To test such networks, we collected all available networks from SNAP (11) with up to 1,000,000 edges where Leiden’s partition gives relatively low modularity values (<0.65), indicating that they are potentially noisier. For these additional networks, TAU led to 1.9% improvement in modularity on average with respect to the Leiden solution, compared to 0.4% improvement on average in the previous set of networks (Supplementary Table S3 and Supplementary Fig. S7).

Since our experiments on synthetic data revealed that TAU consistently finds partitions that are more similar to ground truth communities compared to Leiden, we tried to see if we can replicate these results also on real-life data. Treating real node attributes as ground truth community labels is known to be problematic (18), as it simultaneously tests the meta-data’s relevance and the algorithm’s performance, with no ability to differentiate between the two. Nevertheless, we examined the similarity between the uncovered partitions and known communities for SNAP data sets that were annotated with “ground-truth” communities based on available meta-data: Email, Wiki, DBLP, Amazon, Youtube, and LiveJournal. The results are provided in Supplementary Figs. S5 and S6 and demonstrate the advantage of TAU over Leiden. In particular, in four of the cases, differences were significant according to a Wilcoxon rank sum test as marked in the figure.

### Biological networks

**Fig. 5.**
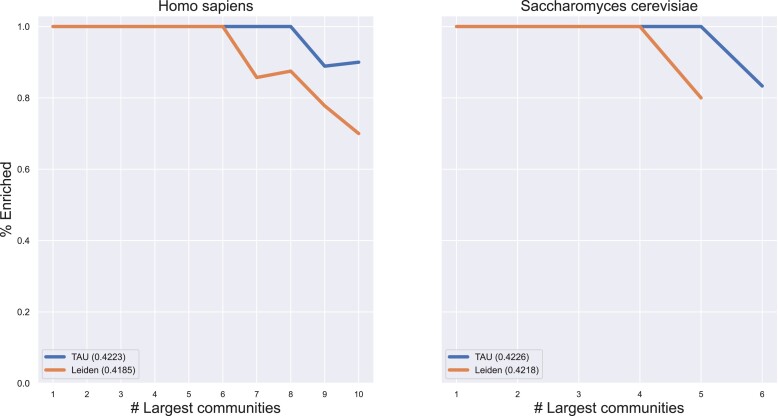
Comparative assessment on biological networks. Modularity values for each method are shown in the legend of each panel.

Last, we wished to assess TAU’s utility for analyzing biological data. To this end, we downloaded the two most comprehensive STRING (14) (version 11.5) protein–protein physical interaction networks: *H. sapiens* and *S. cerevisiae*. We applied TAU and Leiden to the networks and performed functional enrichment analysis for each of the detected communities using GOATOOLS (19) with default settings, focusing on the biological process sub-ontology of the gene ontology (GO). This method compares the set of proteins in a community to each GO term and carries out a statistical test to determine if the term is significantly enriched for the community proteins. True protein communities in the protein–protein interaction networks should include proteins that have similar functional roles, and, as such, they are expected to be GO-enriched. Thus, enrichment analysis could serve as a quality measure for the network partition. For each network, we filtered out communities with less than five proteins and tested the percent of GO-enriched communities while controlling the number of examined largest communities. For both networks, the largest communities found by TAU and Leiden were enriched. However, TAU detected more enriched communities compared to Leiden across the examined range. In addition, we computed the modularity of the identified partitions and found that, similar to previous results, TAU consistently outperformed Leiden. These results are summarized in Fig. 5 and Supplementary Table S2.

## Conclusions

We presented TAU, an algorithm for modularity optimization that efficiently explores the clustering solution space to avoid being trapped in local optima. We applied TAU to a collection of real and synthetic networks and showed that it consistently outperforms Leiden, the current state-of-the-art method, both in terms of modularity value and similarity to ground-truth communities. We found that TAU is particularly effective when the fraction of inter-community connections is large and therefore partition to communities is more challenging—a common characteristic of many complex systems.

For practical use, we also benchmarked TAU when run with a smaller population size on a dual-core CPU and demonstrated its utility also in this scenario. Our approach is defined in a general manner and could potentially be applied to other clustering scenarios such as hierarchical clustering. For example, TAU could be adjusted to optimize Dasgupta’s cost function (20) by replacing the modularity objective with Dasgupta’s objective and using an appropriate agglomerative clustering method.

## Supplementary Material

pgad180_Supplementary_Data

## Data and availability

All data sets are available at (11–14) (see Benchmark data for further information). The code for TAU is freely available at https://github.com/GalGilad/TAU.
