# Supplementary material for: From Leiden to Tel-Aviv University (TAU): exploring clustering solutions via a genetic algorithm
Source: PNAS Nexus. 2023 Jun 1;2(6):pgad180. doi: 10.1093/pnasnexus/pgad180 (PMC10244004; doi:10.1093/pnasnexus/pgad180)
Supplement: pgad180_Supplementary_Data [file pgad180_supplementary_data.pdf]

Supporting Information for

From Leiden to Tel-Aviv University (TAU): exploring clustering solutions via a genetic algorithm

Gal Gilad and Roded Sharan

Roded Sharan
E-mail: roded@tauex.tau.ac.il
Gal Gilad
E-mail: galgilad@mail.tau.ac.il

This PDF file includes:

Figs. S1 to S7
Tables S1 to S3

Graph	Nodes	Edges	Modularity	
			TAU	Leiden-60
Email	776	8,865	.460462	.460450
Astro-ph	16,706	121,251	.74619	.745090
As-22july06	22,963	48,436	.679222	.678743
CAIDARouterLevel	192,244	609,066	.872794	.872504
DBLP	317,080	1,049,866	.840592	.839555
Amazon	334,863	925,872	.93473	.934417
Youtube	1,134,890	2,987,624	.735965	.734242
Wiki	1,281,369	2,988,992	.860274	.859034
LiveJournal	3,997,962	34,681,189	.776072	.774758

Table S1. Performance on real data. Modularity of partitions produced by TAU and Leiden-60.

Graph	Nodes	Edges	# Communities		Modularity		Solution similarity
			TAU	Leiden	TAU	Leiden	
Homo sapiens	18,384	995,916	13	13	.4223	.4185	.59
Saccharomyces cerevisiae	5,966	240,380	7	6	.4226	.4218	.80

Table S2. Performance on biological networks. Solution similarity is computed by the Jaccard similarity between the partitions produced by TAU and Leiden.

Graph	Nodes	Edges	Modularity	
			TAU	Leiden
Wiki-Vote	7,115	103,689	0.430638	0.423664
Enron	36,692	183,831	0.633802	0.626143
Gnutella	62,586	147,892	0.541172	0.523171
Epinions	75,879	508,837	0.458663	0.457742
Slashdot	82,140	549,202	0.34309	0.333732

Table S3. Performance on low-modularity (< 0.65) networks.

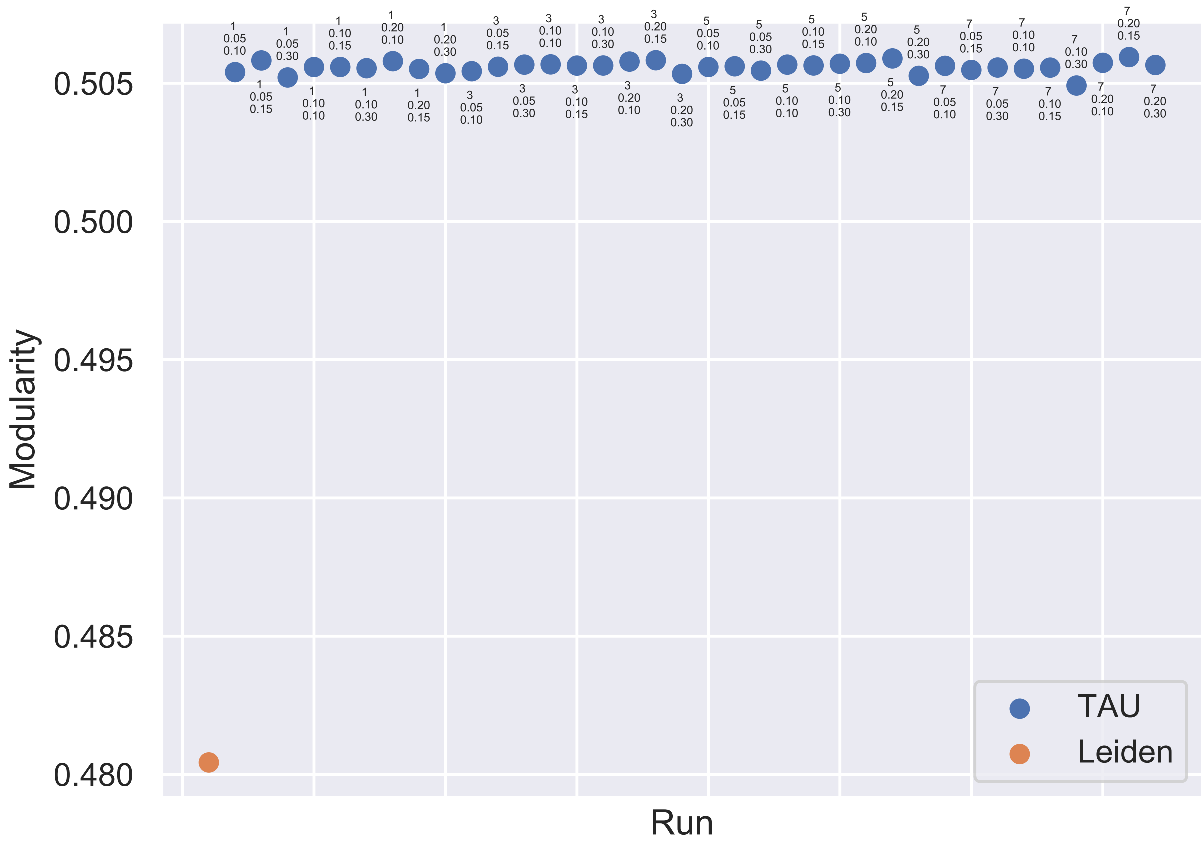

Fig. S1. Parameter tuning - synthetic graph. Annotations are the parameter values that were assigned to (from top to bottom) $power, p\_elite$ and $p\_immigrants$ in each run.

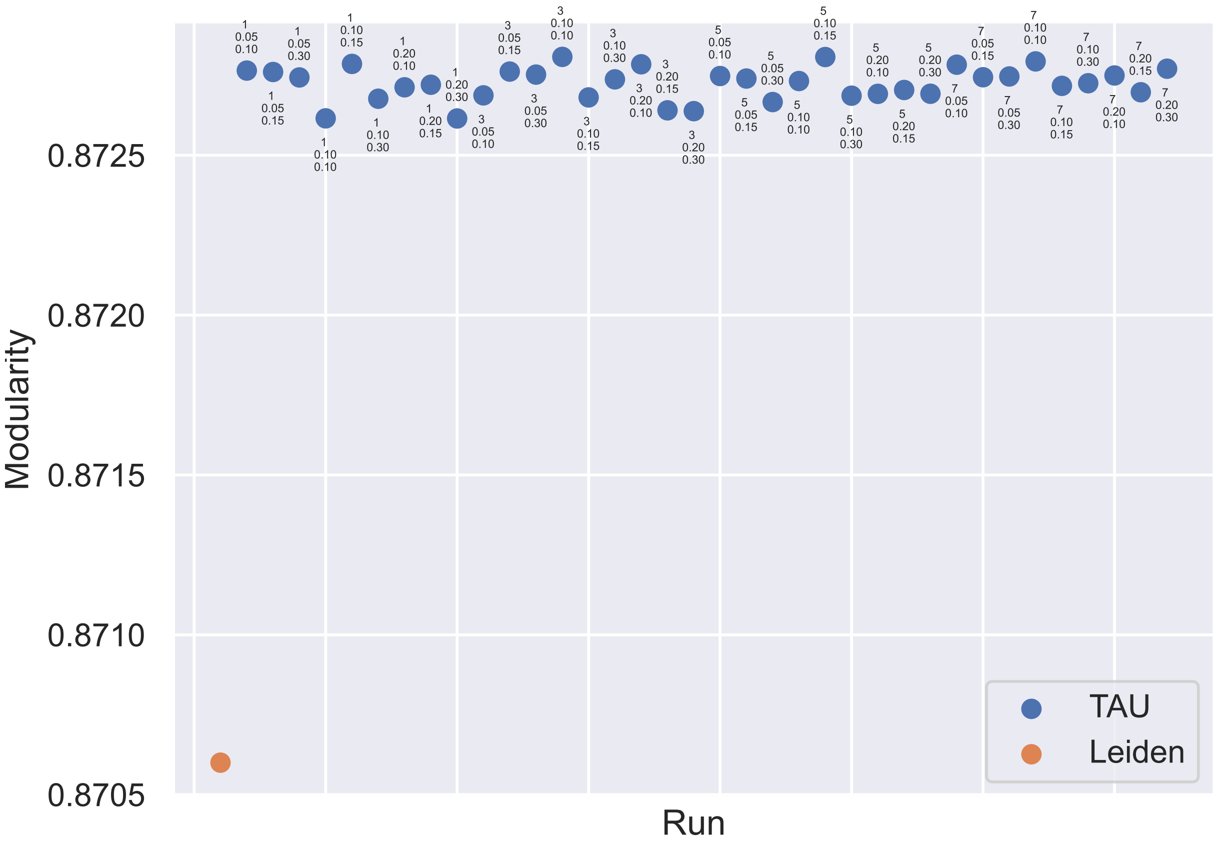

Fig. S2. Parameter tuning - CAIDARouterLevel graph. Annotations are the parameter values that were assigned to (from top to bottom) $power$, p_elite and $p\_immigrants$ in each run.

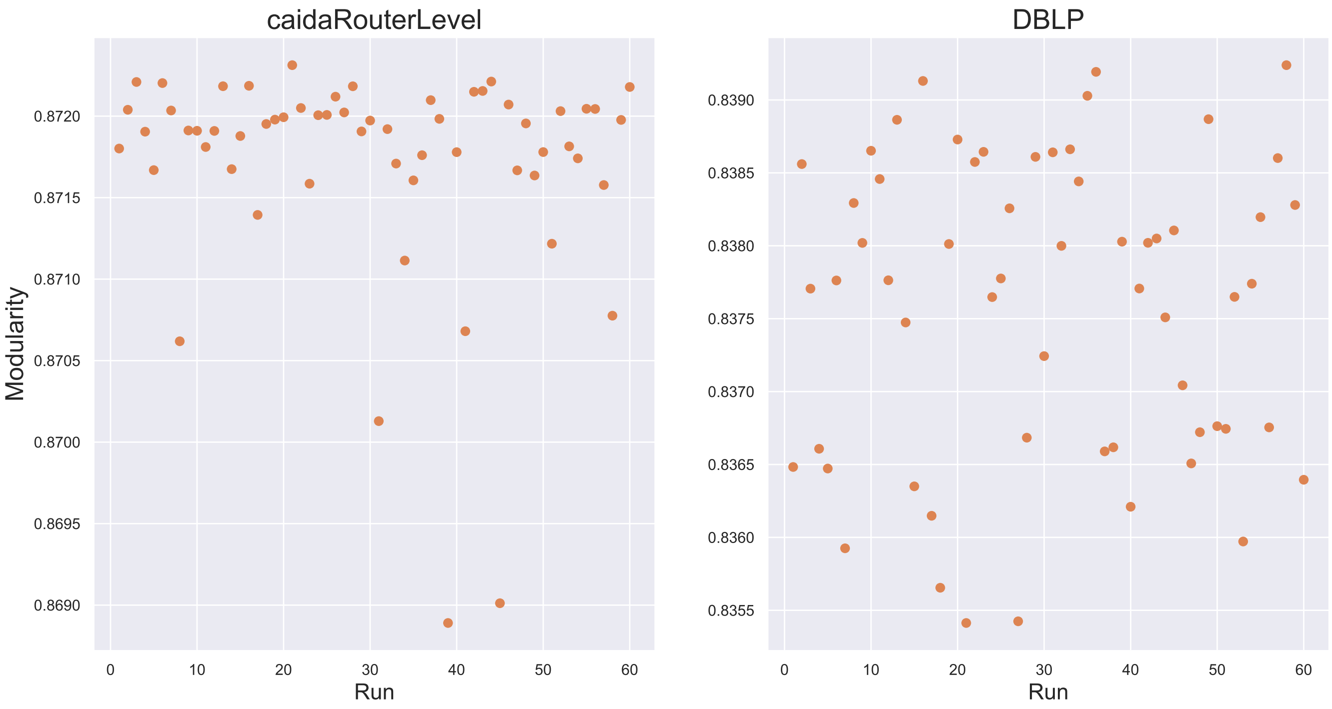

Fig. S3. Leiden performance over 60 runs on caidaRouterLevel and DBLP graphs.

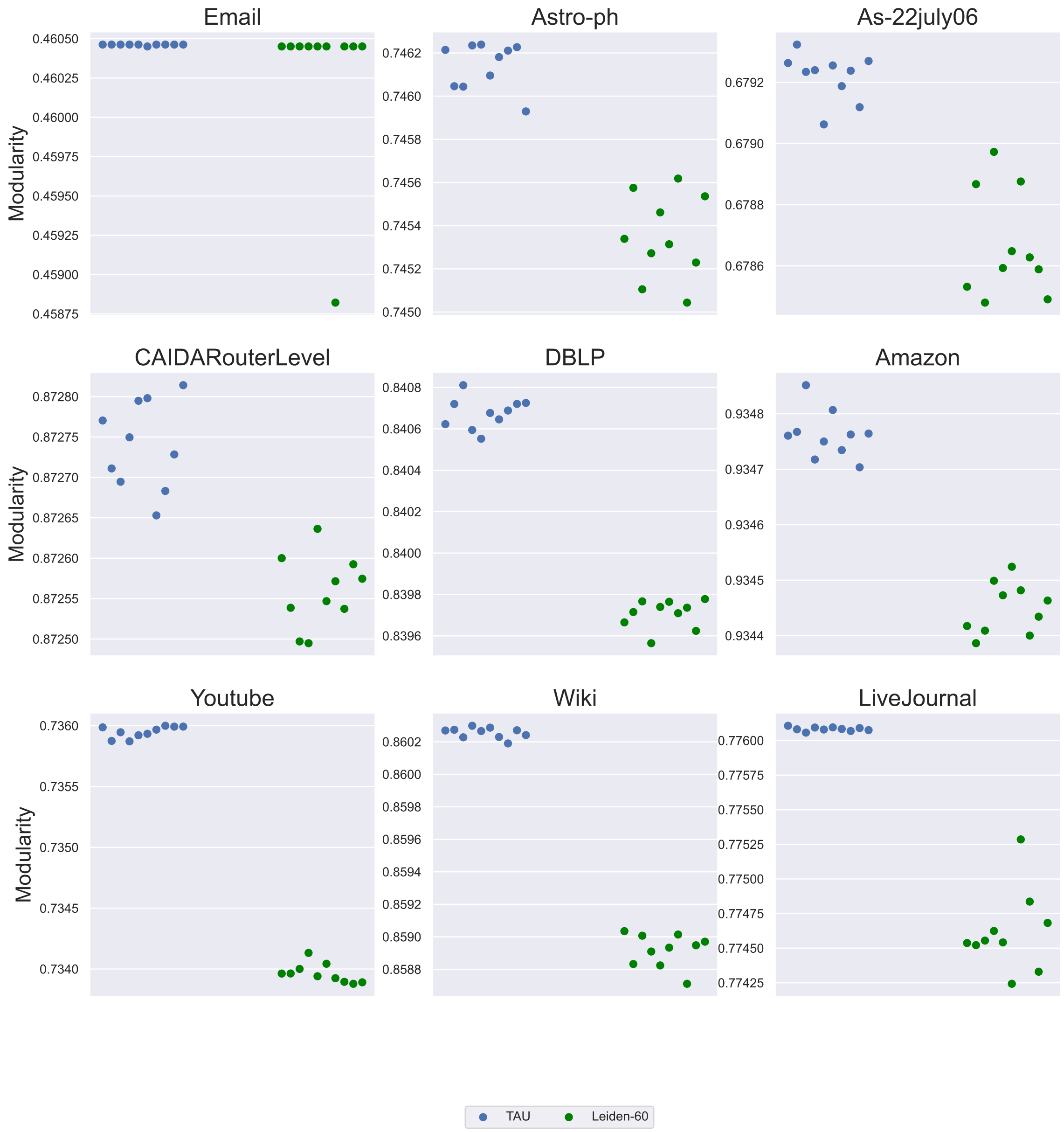

Fig. S4. Comparative assessment on real data. Each dot corresponds to a run of one of the methods - TAU and Leiden-60. We ran each method 10 times. We let Leiden run iteratively for the same amount of time as TAU.

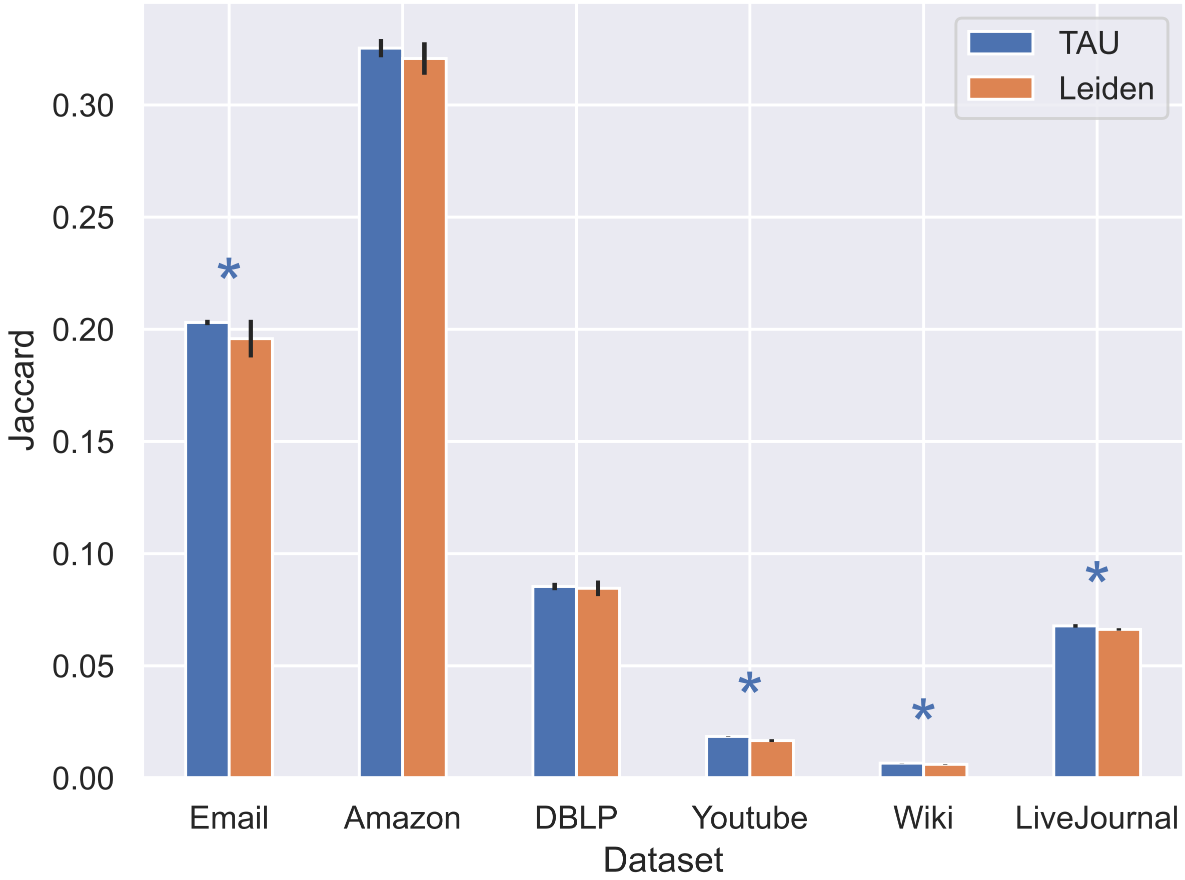

Fig. S5. Similarity to "ground-truth" (Jaccard). Bars represent the mean and standard deviation values of the Jaccard similarity between partitions detected by TAU/Leiden and meta-data based "ground-truth" communities. Results are based on 10 runs for each method. A star denotes a statistically significant difference between TAU and Leiden.

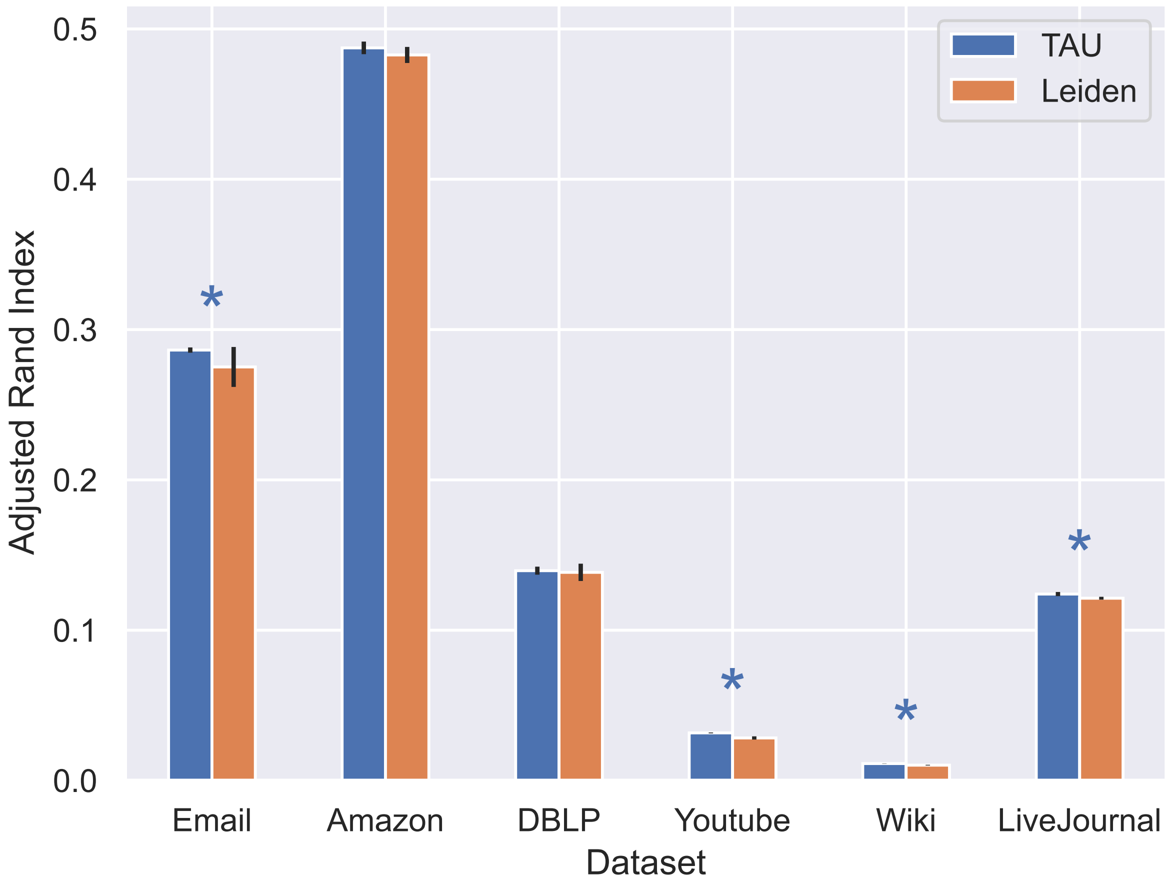

Fig. S6. Similarity to "ground-truth" (Adjusted Rand Index). Bars represent the mean and standard deviation values of the Adjusted Rand Index between partitions detected by TAU/Leiden and meta-data based "ground-truth" communities. Results are based on 10 runs for each method. A star denotes a statistically significant difference between TAU and Leiden.

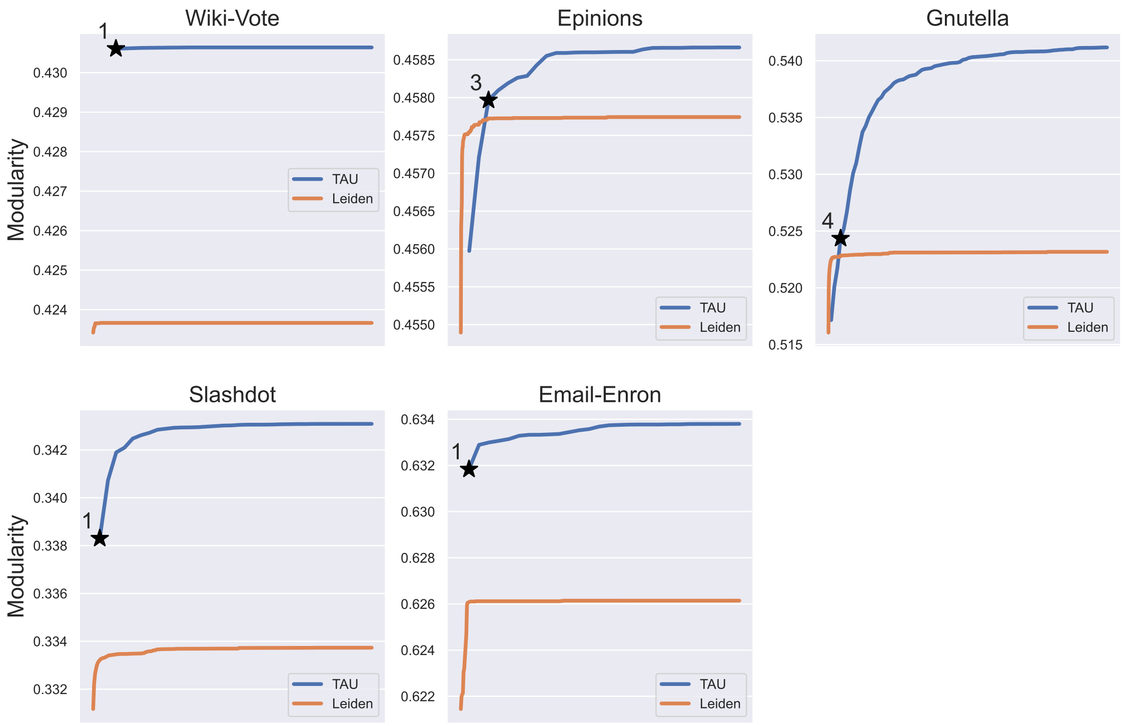

Fig. S7. Performance on low-modularity (< 0.65) networks. The first generation where TAU finds a partition of higher quality compared to the final Leiden partition is marked with a star and the generation's number.
